# Is Nigeria prepared and ready to respond to the COVID-19 pandemic in its conflict-affected northeastern states?

**DOI:** 10.1186/s12939-020-01192-6

**Published:** 2020-05-27

**Authors:** Salman Jidda Tijjani, Le Ma

**Affiliations:** 1 The First Affiliated Hospital, Xi’an Jiaotong University, Xi’an, 710061 China; 2 School of Public Health, Xi’an Jiaotong University Health Science Center, Xi’an, 710061 China; 3 Key Laboratory of Environment and Genes Related to Diseases (Xi’an Jiaotong University), Ministry of Education of China, Xi’an, 710061 China

**Keywords:** COVID-19, Northeastern Nigeria, Humanitarian crisis, Internally displaced persons (IDPs)

## Abstract

Northeastern Nigeria has over the decade suffered from the Boko Haram insurgency and is still in the process of recovery from the complex humanitarian crisis that has displaced and subjected millions of vulnerable children, women and elderly population to poverty, disease outbreaks, hunger and malnutrition. Yet, the conflict-affected states in Northeastern Nigeria is not far away from being the worse-hit by the COVID-19 pandemic if urgent public health preventive measures are not taken to contain the spread of the deadly and highly infectious virus. The question arises, “what is Nigeria doing to tackle the burden of a COVID-19 spread and an ongoing humanitarian crisis?

## Background

Soon after Coronavirus disease-19 (COVID-19) was first reported in an Italian citizen that arrived Nigeria on February 27, 2020, by the Nigerian Center for Disease Control on the 28th January 2020 [1], the National Emergency Operation Centers were immediately activated to level 3 to trace and test all his contacts, and the Presidential Task Force on Covid-19 was inaugurated 3 weeks later [1, 2]. The number of new cases from community transmissions of COVID-19 has been increasing steadily since the index case was first reported [2, 3]. As of 2nd of April, the total confirmed cases of COVID-19 within Nigeria had risen to 184, with 2 (1%) deaths, 20 (11%) discharges, and 162 (88%) cases currently receiving supportive care [3]. Till this date, the total cases reported in the 13 affected states are: Lagos (98), Federal Capital Territory (38), Osun (14), Oyo (8), Akwa Ibom (5), Edo (4), Kaduna (4), Ogun (4), Bauchi (3), Enugu (2), Ekiti (2), Benue (1), and Rivers (1) [3]. Of the 184 confirmed cases, 93 (51%) have travel history to high risk countries, 35 (19%) are contacts of known confirmed cases, and 56 (30%) cases have inconsistent epidemiological information [3].

Although the case fatality rate is very low as at 2nd of April [3], there are public health concerns that the community transmissions of COVID-19 in Nigeria may exponentially rise in the coming weeks, and wreak havoc to nearly seven million people in dare need of humanitarian assistance across the conflict-affected Northeastern part of Nigeria [4–6], which has since seen its first case of COVID-19 reported in Bauchi state on the 24th of March, 2020 [2]. As people continue to travel freely within, and between the Northeastern states, it is only a matter of time before COVID-19 spreads to the rest of the conflict-affected states of Northeastern Nigeria (Borno, Yobe, Gombe, Adamawa, and Taraba states) [7, 8]. Priority high-risk areas to look out for a likely COVID-19 massive spread includes; Borno, Adamawa, and Yobe states, due to lack of laboratory centers for testing COVID-19 in those areas, Internally Displaced Persons (IDPs) presence, food insecurity, limited public health, and primary care services amidst other complex humanitarian challenges [2, 5, 7].

## Main text

### Priority gaps and challenges

Two factors are the likely reasons to contribute to the anticipated spread of COVID-19 in Northeastern Nigeria.

First, it is probable that the concentrated populations of IDPs (in camps, camp-like settings and host communities) experiencing humanitarian crises are at high risk of contracting COVID-19 due to; influx of new arrivals of displaced populations from neighboring villages, and towns affected with COVID-19, frequent displacements due to the Boko Haram insurgency, high density population in urban centers, lack of essential needs (such as food, water, shelter, health, livelihoods, and non-food items), overcrowding, poor housing, lack of access to potable water, insufficient sanitation and hygiene facilities, traditional beliefs and practices, and inadequate awareness of public health preventive measures [5, 7, 9, 10]. More so, practicing social distancing, quarantine, isolation, infection, prevention, and control measures are difficult to perform in complex humanitarian settings with pre-existing structural challenges such as the overwhelming concentrations in IDPs camps, and camp-like settings seen in host communities [7, 9, 10]. Similarly, imposing a complete lock down to the pre-existing movement restrictions in some areas of the conflict-affected states of the Northeastern region will compound the existing humanitarian needs, and operational challenges facing the affected areas [7, 10].

The second factor which is anticipated to make COVID-19 response very challenging in this vulnerable population is the high prevalence of poverty, double burden of endemic infectious, and non-communicable diseases [10] which has been on the increase since the emergence of the Boko Haram insurgency that has devastated the Northeastern part of Nigeria [7]. Dubbed by the World Bank as the poverty capital of the world, and Africa’s most populous nation [4], Nigeria, its Northeastern region in particular, has experienced the highest prevalence of poverty, malnutrition, anemia, malaria, and has repeatedly over the past decade seen the emergence, and re-emergence of endemic diseases such as; cholera, measles, Lassa fever, meningitis, HIV and TB [5]. It is therefore expected that the humanitarian situation in this region will worsen particularly due to COVID-19 [7], the double burden of diseases, and conflict overstretching the primary healthcare facilities [10], which underlines an urgent need for rapid action in order to meet the current projections of people in dare need of humanitarian assistance [2, 5, 7]. Given these increased vulnerabilities in humanitarian settings, the United Nations emphasized that diverting funds from addressing the unmet humanitarian needs of the most vulnerable people (especially the ultra-poor, children and elderly populations) in this pandemic era, would create a breeding ground for COVID-19 to thrive and also lose gains made in the fight against already existing infectious disease epidemics, endemics and other public health emergencies [7].

The question arises, “Is Nigeria, in particular, the Northeastern region, prepared and ready to tackle yet another epidemic of COVID-19? What is Nigeria presently doing to stem the impact of a community transmission of the highly infectious disease-COVID-19, in already vulnerable and very challenging humanitarian context?

### Ongoing response

The answer to this, is, the Federal Government of Nigeria is better prepared than ever before [11, 12]. Nigeria was among the first countries to recognize the risk and start planning ahead of the epidemic curve 1 week after China first reported the cases of COVID-19 [12]. So far, the country have recorded giant strides in the fight against COVID-19 by drawing on successes, and lessons learnt from controlling previous epidemics (such as Ebola and Polio), ongoing epidemics of Lassa fever [11–13], followed by significant financial investments into preparedness, and surveillance from the Federal Government, and the newly formed Nigerian Private Sector Coalition Against COVID-19 [14]. The Federal Government of Nigeria through the Presidential Task Force on COVID-19, Federal Ministry of Health and Nigeria Center for Disease Control, have been working closely in collaboration with relevant ministries, departments, agencies, partners, and other stakeholders to coordinate and review the national response strategies and implementation activities, on a daily basis in order to effectively contain the spread of COVID-19 [2, 8, 14].

Till date, several non-pharmaceutical measures have been employed to limit the importation of new cases, and control local transmissions. These measures include; staying at home, travel bans to, and from high-risk countries with community transmissions of COVID-19, border controls, deployment of rapid response teams to all affected states, state-level training and capacity building of health personnel on; infection, prevention and control; case management, intensified risk communication, community engagement, heightened surveillance, field epidemiological investigations, rapid identification of suspected cases, isolation, diagnosis, contact tracing, monitoring and follow-up of persons of interests [2, 7, 15]. Furthermore, social and religious gatherings were banned temporarily, schools and businesses were closed, restriction of movements and partial lock down were instituted in the Federal Capital Territory, Lagos, and Ogun states [8, 15]. The country has also established and expanded the diagnostic capacity for COVID-19 in seven laboratories in 2 months with plans to increase to 13 more locations in the coming weeks [2, 3].

In addition, the Federal Government of Nigeria expanded its social safety net, and welfare programmes in its efforts to reduce poverty, and mitigate impacts of the unintentional partial-lock down on the livelihoods of the poor, and vulnerable households in affected states [8]. Food rations, food vouchers, conditional cash transfers, and other forms of palliatives targeted vulnerable, and socially disadvantaged members of the affected communities [8]. In Borno state (epicenter of the humanitarian crisis in Northeastern Nigeria), sensitization campaigns on handwashing were followed by the distribution of soaps to more than one hundred thousand internally displaced persons [16] in consistent with Peterson and colleagues’ findings establishing the relationship between distribution of soaps in humanitarian settings with increased handwashing by over 30% [17].

## Conclusions

The United Nations has recently called on all donors, partners and stakeholders to properly fund and support their Global Humanitarian Respond Plan to fight the common threat of COVID-19, by providing laboratory materials for testing, personal protective equipment for health workers, medical equipment to treat the sick, supplying water, and installing handwashing stations in camps and settlements [7]. It is of extreme importance the Nigerian Government and stakeholders, continue to sustain and step up their commitment for mobilizing more public health resources to be better prepared and more proactive than ever to scale up COVID-19 interventions and future epidemic preparedness and public health preventive measures for people affected by humanitarian crisis. More importantly, humanitarian actors, local, and international authorities are recommended to adopt the use of evidenced-based public health guidelines, such as the Sphere handbook, to guide their response to the COVID-19 pandemic in similar humanitarian context [18].

## Data Availability

Not applicable.
